# Switching within the class of IL‐17 inhibitors for the treatment of plaque psoriasis: A real‐world retrospective study

**DOI:** 10.1111/ajd.14396

**Published:** 2024-12-12

**Authors:** Ioannis‐Alexios Koumprentziotis, Natalia Rompoti, Irene Stefanaki, Charitomeni Vavouli, Marina Papoutsaki, Maria Politou, Alexander Stratigos, Electra Nicolaidou

**Affiliations:** ^1^ 1st Department of Dermatology‐Venereology, Medical School National and Kapodistrian University of Athens, "A. Sygros" Hospital for Skin and Venereal Diseases Athens Greece

**Keywords:** bimekizumab, biologics, Il‐17 inhibitors, intraclass switch, psoriasis

Dear Editor,

The blockade of the interleukin (IL)‐17 signalling pathway has revolutionized the treatment of psoriasis with agents such as ixekizumab, and secukinumab (IL‐17A inhibitors), brodalumab (IL‐17 receptor inhibitor) and bimekizumab (IL‐17A and IL‐17F inhibitor). Still, primary or secondary failure is observed in some patients forcing us to decide whether we will proceed with an agent that is not an IL‐17 inhibitor or perform an intraclass switch.^1^ The available literature on switching between IL‐17 inhibitors is generally limited and up to date there are no real‐world studies reporting on patients switching to the most recently approved IL‐17A/F inhibitor, bimekizumab.^2^, ^3^, ^4^, ^5^, ^6^ Therefore, we aimed to assess whether switching from an IL‐17 inhibitor to another (including switching to bimekizumab) is an effective and safe option in a real‐world setting.

We conducted a single‐centre, retrospective study of adult patients with moderate‐to‐severe plaque psoriasis that discontinued an IL‐17 inhibitor and were subsequently switched to another agent within the same class. The follow‐up period was 104 weeks, with the exception of those that switched to bimekizumab who were followed for up to 52 weeks due to bimekizumab's recent approval in our country.

In total, 61 patients (40 males, 21 females) were included in the study and their baseline characteristics are presented in Table 1. Twenty‐one patients had switched to bimekizumab, 16 to brodalumab, 18 to ixekizumab and 6 to secukinumab. The reason for IL‐17 inhibitor discontinuation was primary failure (14 patients), secondary failure (44 patients) and adverse events (AEs) (3 patients). Primary failure was defined as insufficient response (patients not achieving psoriasis area severity index (PASI) 50 at weeks 12–16), according to the medication dosing scheme. Secondary failure was defined as the loss of response of PASI >50% of initial value in a patient who had previously achieved PASI 50 response at week 12–16.

**TABLE 1:** Patients' baseline characteristics at the time of drug switching.

	Bimekizumab	Brodalumab	Ixekizumab	Secukinumab
Number of patients	21	16	18	6
Female, *n* (%)	8 (38.1%)	2 (12.5%)	8 (44.4%)	3 (50%)
Age at drug initiation (years), mean (range), (SD)	50.1 (13–72) (13.56)	49.69 (27–72) (12.53)	48.61 (25–79) (14.72)	56.33 (48–79) (11.86)
Age at disease onset (years), mean (range) (SD)	31.47 (6–58) (16.9)	30.06 (15–53) (12.83)	26.53 (14–58) (11.34)	35 (20–59) (13.57)
Disease duration (years), mean (range) (SD)	19.89 (3–43) (10.8)	19 (2–32) (9.52)	23.88 (6–46) (11.63)	21 (6–39) (10.56)
Baseline PASI, mean (SD)	10.3 (8.34)	8.98 (5.73)	5.57 (4.86)	5.43 (5.15)
BMI (kg/m^2^), mean (SD)	32.46 (6.72)	29.85 (4.72)	30.37 (5.38)	31.02 (8.59)
Comorbidities, *n* (%)	13 (61.9%)	11 (68.8%)	12 (66.7%)	4 (66.7%)
Cardiovascular disorders, *n* (%)	9 (42.9%)	6 (37.5%)	9 (50%)	1 (16.7%)
Metabolic disorders, *n* (%)	8 (38.1%)	5 (31.3%)	8 (44.4%)	4 (66.7%)
Psychological/psychiatric disorders, *n* (%)	1 (4.8%)	2 (12.5%)	3 (16.7%)	1 (16.7%)
Concomitant PsA, *n* (%)	10 (45.5%)	1 (6.3%)	6 (33.3%)	5 (83.3%)
Scalp psoriasis, *n* (%)	9 (42.9%)	7 (43.8%)	9 (50%)	5 (83.3%)
Palmoplantar plaque psoriasis, *n* (%)	6 (28.6%)	7 (43.8%)	7 (38.9%)	4 (66.7%)
Genital psoriasis, *n* (%)	3 (14.3%)	6 (37.5%)	6 (33.3%)	4 (66.7%)
Nail psoriasis, *n* (%)	7 (33.3%)	9 (56.3%)	12 (66.7%)	5 (83.3%)
Previous therapy with				
≥2 biologics, *n* (%)	18 (85.7%)	8 (50%)	12 (66.7%)	4 (66.7%)
Anti‐TNFa, *n* (%)	14 (66.7%)	5 (31.3%)	8 (44.4%)	4 (66.7%)
IL‐12/23 inhibitor, *n* (%)	6 (28.6%)	4 (25%)	6 (33.3%)	1 (16.7%)
Apremilast, *n* (%)	8 (38.1%)	7 (43.8%)	5 (27.8%)	2 (33.3%)
IL‐23 inhibitor, *n* (%)	1 (4.8%)	1 (6.3%)	0 (0%)	0 (0%)
Bimekizumab, *n* (%)	0 (0%)	0 (0%)	0 (0%)	1 (16.7%)
Brodalumab, *n* (%)	8 (38.1%)	0 (0%)	9 (50%)	5 (83.3%)
Ixekizumab, *n* (%)	7 (33.3%)	2 (12.5%)	0 (0%)	0 (0%)
Secukinumab, *n* (%)	6 (28.6%)	14 (87.5%)	9 (50%)	0 (0%)
Reasons of discontinuation of directly previous IL‐17 inhibitor				
Primary failure, *n* (%)	4 (19%)	5 (31.3%)	5 (27.8%)	0 (0%)
Secondary failure, *n* (%)	17 (80.9%)	10 (62.5%)	12 (66.7%)	5 (83.3%)
Adverse events, *n* (%)	0 (0%)	1 (6.3%)	1 (5.6%)	1 (16.7%)

Regarding patients that switched to bimekizumab, a PASI 75/90 response was observed in 78.6/64.3% of the evaluated patients at 24 weeks while all three patients reaching 52 weeks of treatment had a clear skin (Table 2). After switching to brodalumab, PASI75/90 at 24 and 52 weeks was observed in 75/58.3% of the evaluated patients with 66.7% achieving great control of their disease with a PASI≤1 score at week 104. For patients that switched to ixekizumab, a PASI 75/90 was reached by 75/68.8% and 69.2/61.5% of patients at 24 and 52 weeks respectively. After 104 weeks, 71.4% achieved PASI≤1. Of the six patients switching to secukinumab, PASI75/90 response was observed in 83.3/50% after 24 weeks and 75/50% after 52 weeks.

**TABLE 2:** Clinical responses of patients with available data that were evaluated at each time point after IL‐17 intraclass switching.

	Bimekizumab	Brodalumab	Ixekizumab	Secukinumab
Week 16				
PASI 75	13/16 (81.3%)	8/12 (66.67%)	12/16 (75%)	4/4 (100%)
PASI 90	10/16 (62.5%)	6/12 (50%)	11/16 (68.8%)	3/4 (75%)
PASI 100	10/16 (62.5%)	6/12 (50%)	11/16 (68.8%)	3/4 (75%)
PASI≤3	11/16 (68.8%)	9/12 (75%)	16/16 (100%)	4/4 (100%)
PASI≤1	11/16 (68.8%)	7/12 (58.3%)	11/16 (68.8%)	3/4 (75%)
Week 24				
PASI 75	11/14 (78.6%)	9/12 (75%)	12/16 (75%)	5/6 (83.3%)
PASI 90	9/14 (64.3%)	7/12 (58.3%)	11/16 (68.8%)	3/6 (50%)
PASI 100	9/14 (64.3%)	7/12 (58.3%)	9/16 (56.3%)	3/6 (50%)
PASI≤3	11/14 (78.6%)	9/12 (75%)	14/16 (87.5%)	5/6 (83.3%)
PASI≤1	9/14 (64.3%)	7/12 (58.3%)	11/16 (68.8%)	3/6 (50%)
Week 52				
PASI 75	3/3 (100%)	9/12 (75%)	9/13 (69.2%)	3/4 (75%)
PASI 90	3/3 (100%)	7/12 (58.3%)	8/13 (61.5%)	2/4 (50%)
PASI 100	3/3 (100%)	7/12 (58.3%)	8/13 (61.5%)	2/4 (50%)
PASI≤3	3/3 (100%)	9/12 (75%)	12/13 (92.3%)	3/4 (75%)
PASI≤1	3/3 (100%)	9/12 (75%)	8/13 (61.5%)	3/4 (75%)
WEEK 104				
PASI 75	NA	6/6 (100%)	5/7 (71.4%)	NA
PASI 90	NA	4/6 (66.7%)	5/7 (71.4%)	NA
PASI 100	NA	4/6 (66.7%)	5/7 (71.4%)	NA
PASI≤3	NA	6/6 (100%)	5/7 (71.4%)	NA
PASI≤1	NA	4/6 (66.7%)	5/7 (71.4%)	NA

During observation, three patients (2 receiving bimekizumab, and 1 receiving ixekizumab) experienced fungal infections (2 intertriginous infections and 1 stomatitis) that were promptly managed while no other serious AEs occurred. None of the three patients that discontinued the previous IL‐17 inhibitor due to AEs experienced any similar AEs after switching.

Our findings are similar to reports from other studies that evaluated IL‐17 intraclass switching.^2^, ^3^, ^4^, ^5^, ^6^ Regarding secukinumab and ixekizumab, our findings are within the reported range of real‐world studies^7^, ^8^ while for brodalumab, better clinical outcomes with more patients achieving PASI90/100 at 24 and 52 weeks were reported by a previous study by our centre.^9^ Concerning bimekizumab use after failure of a previous IL‐17 inhibitor, relevant data were reported in the BE RADIANT clinical trial.^10^ Patients that received secukinumab initially and did not meet PASI90 (PASI90 non responders) were switched to bimekizumab, with 79% achieving PASI90 after 48 weeks of treatment without any additional safety findings.^10^ Overall, even in clinical trial settings, IL‐17 intraclass switching seemed to be a successful approach for the management of psoriasis.^10^ With concern to safety, the AEs that occurred were within the range reported in clinical trials. The crucial role of IL‐17 in the immunity against fungal pathogens may be highlighted with bimekizumab, dually inhibiting IL17‐A and F, exhibiting relatively higher risk of fungal infections compared to the other agents.

The main limitation of our study is the relatively small number of patients, especially for the bimekizumab cohort, with only three patients reaching the 52‐week timepoint, thus not allowing for proper assessment of the long‐term efficacy of intraclass switching. However, our study has a long follow‐up period of 104 weeks for brodalumab and ixekizumab, which is longer compared to other studies on IL‐17 intraclass switch^2^, ^3^, ^4^, ^5^, ^6^ and, furthermore, it included patients switching to bimekizumab, which, to our knowledge, has not been reported in real‐world settings before.

In conclusion, our findings are in accordance with the available literature and suggest that performing an intraclass switch among IL‐17 inhibitors is a generally effective and safe option. Further studies are needed to draw firmer conclusions.

## FUNDING INFORMATION

No funding was received for this particular manuscript.

## CONFLICT OF INTEREST STATEMENT

I.‐A. Koumprentziotis: No conflict of interest. N. Rompoti: Honoraria for lectures from AbbVie, Genesis Pharma, Janssen, LEO, Lilly Pharmaserve, Novartis, UCB and support for scientific congress attendance from. AbbVie, Janssen, LEO, Lilly Pharmaserve, Novartis, UCB. ‐I. Stefanaki: Honoraria from AbbVie, Genesis, Janssen, LEO Pharma and Novartis. C. Vavouli: Honoraria for advisory boards from LEO Pharma, Galenica, honoraria for lectures from LEO Pharma, Genesis, Novartis, and support for congress attendance from LEO Pharma, Genesis, Novartis, and Janssen. ‐M. Papoutsaki: Honoraria for advisory boards and lectures from Janssen, LEO. Pharma, MSD, Genesis Pharma, Pfizer, Novartis, AbbVie, UCB, and support as investigator in clinical studies from AbbVie, Novartis, LEO Pharma, Janssen. ‐M. Politou: Honoraria for advisory boards from AbbVie, Novartis, Genesis, LEO. Pharma, honoraria for lectures from AbbVie, Janssen, Novartis, Genesis, UCB, LEO. Pharma. A. Stratigos: Honoraria for advisory boards, lectures, and support for international congress attendance from Bristol‐Myers Squibb, Novartis, Merck Sharp & Dohme, Sanofi, and research support from Hoffmann‐La Roche. E. Nicolaidou: Honoraria for advisory board, lectures, and support for scientific meeting attendance from Janssen, LEO Pharma, Novartis, UCB, Lilly Pharmaserve, Gelgene/Genesis Pharma, Galenica, Amgen.

## ETHICS STATEMENT

The patients in this manuscript have given written informed consent to publication of their case details. Ethical approval was given by the hospital ethics committee.

## Data Availability

The data that support the findings of this study are available from the corresponding author upon reasonable request.
